# Effects of different physical forms of concentrate on performance, carcass characteristics, and economic analysis in hanwoo steers

**DOI:** 10.1186/2055-0391-56-9

**Published:** 2014-07-28

**Authors:** Sung Il Kim, Bo cheon Seo, In Surk Jang, Ouk Kim, Chang Bon Choi, Keun Ki Jung

**Affiliations:** Department of Animal Science, Gyeonbuk Provincial College, Yecheong-eup, 757-807 Korea; Gyeongnam National University of Science and Technolony, Jinju, 757-803 Korea; Department of Animal Science, Dong-A University, Busan, 602-714 Korea; Department of Biotechnolony, Yeungnam University, Gyeongsan, 712-749 Korea; Moksan Hanwoo Reserch Institute, 108-7, Bujeok-ri, Apryang-myun, Gyeongsan, Gyeongsangbuk-do, 712-821 Korea

**Keywords:** Hanwoo steers, Flaked concentrate, Grounded concentrate, Economic performance

## Abstract

This study was performed to investigate the effects of different forms of concentrate fed to Hanwoo steers on performance, carcass characteristics, and economic performance. Forty-two Hanwoo steers (average age of 5.1 ± 0.8 mo. with body weight of 147.05 ± 10.85 kg) were randomly allotted into FC (animals fed flakes for entire experimental period) and GC (animals fed grounded concentrate during growing and fattening phases followed by flaked concentrate during finishing phase) groups for 758 d after reaching an age of 30.0 ± 0.82 mo. There was no difference in body weight (BW) or ADG between the treatments until fattening (15 ~ 22 mo.) phase. However, by finishing phase (23 ~ 30 mo.), the GC group (739.24 kg BW and 0.67 kg ADG) showed greater (P < 0.05) BW and ADG than the FC group (702.93 kg BW and 0.59 kg ADG). Steers in the GC group also showed greater (P < 0.05) BW and ADG than the FC group throughout the entire experimental period (5 ~ 30 mo.). There was no significant difference in carcass weight or backfat thickness between the treatments. *M. Longissimus dorsi* area of the GC group (91.00 cm^2^cm^2^) was greater (P < 0.05) than that of the FC group (83.59 cm^2^). Marbling score and percentage of 1^++^ meat quality grade were 14.0 and 48.0% higher in the GC group compared to the FC group. There was no significant difference in physicochemical characteristics, including moisture and crude protein levels, between the treatments. Gross income per head excluding operating expenses was 59.3% greater in the GC group (1,647,512 won) compared to the FC group (1,034,343 won).

## Background

The current trend in feeding systems for Hanwoo steers in Korea involves administration of animal feeds in the form of pellets or flakes rather than as grounded feeds. Pellets are formed by grounding and compressing raw ingredients into the shape of a pellet while flakes are processed using high heat and pressure. Hanwoo steers are fed grain-oriented compound feeds to increase their intake and efficiency [1]. However, production costs of Hanwoo farms are rapidly increasing due to skyrocketing prices of grains and animal feeds. As such, there is increasing demand to fortify the competitiveness of the Hanwoo industry by cutting down on production costs, and the most effective method may be simplifying the entire animal feed process. Processing of feedstuffs increases the gelatinization and digestibility of starch, ultimately improving feed efficiency [2]. Gelatinization of starch can be increased by pelleting and flaking by 35% and 50%, repectively [3, 4]. The range of this processing effect and feed efficiency depend on the type of grain, processing method, origin of feed ingredients, and breeding period of cattle [5, 6]. Therefore, it is necessary to appropriately adjust the processing method of feed with regard to the breeding period of Hanwoo steers. To this end, this study investigated the effects of administration of various physical forms of grain feed during different feeding phases on performance, carcass characteristics, and economic performance in Hanwoo steers, thereby reducing production costs with efficient feed processing methods.

## Materials and methods

### Experimental animals and design

Forty-two Hanwoo steers (average age of 5.1 ± 0.8 mo. with body weight of 147.05 ± 10.85 kg) were randomly allotted into FC (animals fed flakes for entire experimental period; growing, fattening, and finishing phases, 5 ~ 30 mo. of age) and GC (animals fed grounded concentrate during growing, 5 ~ 14 mo. of age, and fattening phases, 15 ~ 22 mo. of age, followed by flakes during finishing phase, 23 ~ 30 mo. of age). Hanwoo steers in the FC (18 steers divided into three pens) and GC (24 steers divided into four pens) groups were administered the assigned diets for 758 d after reaching an age of 30.0 ± 0.82 mo. (Table 1). The animals were sacrificed for meat production and parts of the carcass were used for the analysis with consent from the farmers in this study.

**Table 1:** **Feeding regimen of concentrate diet for the entire experiment**

Treatment	Phases		
	Growing^1)^	Fattening^2)^	Finishing^3)^
FC	Flaked & pelleted diet	Flaked & pelleted diet	Flaked & pelleted diet
GC	Ground diet	Ground diet	Flaked & pelleted diet

^1)^Feeding period: 5.1 to 13.9 months of age.

^2)^Feeding period: 13.9 to 22.1 months of age.

^3)^Feeding period: 22.1 to 30.0 months of age.

### Experimental diets

Experimental diets were formulated by an animal feed manufacturing company located in Kimhae, Korea. Roughages used in this study included timothy, alfalfa, and tall fescue. Chemical compositions of the experimental diets are shown in Table 2 (concentrate) and 3 (roughage). Table 4 shows the physicochemical characteristics of corns. Amounts of concentrate and roughages used in the experimental diets were determined by considering the growth stage and nutrient requirements of the animals (Table 5).

**Table 2:** **Chemical composition of concentrate diets**

Composition	Concentrate					
	Growing		Fattening		Finishing	
	Flaked	Ground	Flaked	Ground	Flaked	Flaked
	─────────%, as - fed ──────────					
Moisture	11.78 ± 0.12^1)^	11.82 ± 0.05	12.67 ± 0.06	12.90 ± 0.04	12.59 ± 0.09	12.59 ± 0.09
Crude protein	16.22 ± 0.01	16.33 ± 0.02	15.33 ± 0.04	15.18 ± 0.03	13.26 ± 0.03	13.26 ± 0.03
Crude fat	3.06 ± 0.01	2.63 ± 0.04	3.03 ± 0.03	3.19 ± 0.04	3.17 ± 0.06	3.17 ± 0.06
Crude fiber	11.74 ± 0.25	13.13 ± 0.08	10.15 ± 0.06	10.17 ± 0.10	9.24 ± 0.39	9.24 ± 0.39
Crude ash	5.44 ± 0.06	6.03 ± 0.05	4.89 ± 0.02	5.04 ± 0.06	4.57 ± 0.20	4.57 ± 0.20
NFE	51.76 ± 1.61	50.06 ± 1.32	53.93 ± 1.11	53.52 ± 1.46	57.17 ± 0.95	57.17 ± 0.95
Ca	0.77 ± 0.04	0.66 ± 0.01	0.59 ± 0.01	0.63 ± 0.02	0.54 ± 0.06	0.54 ± 0.06
P	0.45 ± 0.00	0.44 ± 0.00	0.40 ± 0.00	0.43 ± 0.00	0.41 ± 0.02	0.41 ± 0.02
NDF^2)^	29.04 ± 0.36	33.54 ± 0.21	26.06 ± 0.07	25.33 ± 0.07	27.85 ± 1.37	27.85 ± 1.37
ADF^3)^	16.18 ± 0.19	15.63 ± 0.24	14.75 ± 0.10	14.34 ± 0.07	13.58 ± 0.38	13.58 ± 0.38
TDN^4)^	68.0	68.0	70.0	70.0	72.0	72.0

^1)^Means ± standard error.

^2)^Neutral derergent fiber.

^3)^Acid detergent fiber.

^4)^Calculated.

**Table 3:** **Chemical composition of roughages**

Composition	Roughages			
	Timothy hay	Alfalfa hay	Tall fescue straw	Ryegrass straw
	─────%, as-fed basis ────			
Moisture	8.22 ± 0.07^1)^	9.64 ± 0.18	9.76 ± 0.44	7.72 ± 0.04
Crude protein	7.87 ± 0.18	17.79 ± 0.16	7.06 ± 0.59	5.39 ± 0.16
Crude fat	1.89 ± 0.02	1.97 ± 0.02	0.77 ± 0.00	1.10 ± 0.05
Crude fiber	32.77 ± 0.28	27.71 ± 0.32	32.57 ± 1.63	32.34 ± 0.19
Crude ash	6.37 ± 0.12	9.20 ± 0.10	5.97 ± 0.76	5.82 ± 0.07
NFE^2)^	42.88 ± 0.74	33.69 ± 0.97	43.87 ± 2.54	47.63 ± 0.19
Ca	0.28 ± 0.00	1.48 ± 0.02	0.20 ± 0.01	0.36 ± 0.01
P	0.16 ± 0.00	0.22 ± 0.00	0.08 ± 0.01	0.13 ± 0.00
NDF^3)^	59.99 ± 0.30	37.70 ± 0.43	59.42 ± 1.93	60.88 ± 0.10
ADF^4)^	34.36 ± 0.23	30.71 ± 0.68	34.20 ± 1.78	34.45 ± 0.15
TDN^5)^	54.61	53.55	34.18	52.82

^1)^Means ± standard error.

^2)^Nitrogen-free extract.

^3)^Neutral derergent fiber.

^4)^Acid detergent fiber.

^5)^Calculated.

**Table 4:** **Physicochemical characteristics and distribution of particle size in flake and grounded corns**

Items	Flaked corn	Ground corn
Flake thickness^1)^, mm	3.29 ± 0.04^2)^	―
Starch gelatinization^3)^,%	31.93 ± 1.69	―
Density, g/ℓ	506.8 ± 2.0	693.5 ± 2.5
Particle size^4)^,%		
Sieve mesh		
6 ~ 8	―	36.1 ± 2.8
14 ~ 18	―	45.3 ± 4.3
25 ~ 40	―	10.9 ± 1.3
60 ~ 100	―	6.2 ± 0.6
Under 100	―	1.5 ± 0.4

^1)^Measured by vernier calipers.

^2)^Means ± standard error.

^3)^Determined by diastase method.

^4)^Percents retained on screen.

**Table 5:** **Feeding program for Hanwoo steers in the experiment**

Fattening phase	Age in mon.	Body weight range (kg)	Daily gain (kg)	Feeding level (body weight,%)	Concentrate fed (kg/hd/d, as-fed basis)			Roughage fed (kg/hd/d, as-fed basis)		
					Growing	Fattening	Finishing	Timothy hay	Alfalfa hay	Straw
Growing	5	147 ~ 162	0.50	0.90	1.4			1.5	0.5	
	6	162 ~ 188	0.85	1.25	2.0			2.0	1.0	
	7	188 ~ 214	0.85	1.40	2.6			3.0	1.0	
	8	214 ~ 241	0.90	1.50	3.2			3.4	1.0	
	9	241 ~ 268	0.90	1.50	3.6			3.5	1.0	
	10	268 ~ 295	0.90	1.53	4.1			4.0	1.0	
	11	295 ~ 322	0.90	1.55	4.6			4.0	1.0	
	12	322 ~ 351	0.95	1.61	5.2			4.5	0.5	
	13	351 ~ 379	0.95	1.70	6.0			4.5	0.5	
Fattening	14	379 ~ 408	0.95	1.84	3.5	3.5		4.5		
	15	408 ~ 436	0.95	1.96		8.0		3.5		0.5
	16	436 ~ 466	1.00	2.06		9.0				3.0
	17	466 ~ 496	1.00	2.08		9.7				3.0
	18	496 ~ 526	1.00	2.02		10.0				2.5
	19	526 ~ 553	0.90	1.90		10.0				2.3
	20	553 ~ 579	0.85	1.81		10.0				2.0
	21	579 ~ 604	0.85	1.73		10.0				1.5
	22	604 ~ 628	0.80	1.66		10.0				1.5
Finishing	23	628 ~ 649	0.70	1.51			9.5			1.3
	24	649 ~ 667	0.60	1.39			9.0			1.2
	25	667 ~ 682	0.50	1.35			9.0			1.2
	26	682 ~ 696	0.45	1.25			8.5			1.2
	27	696 ~ 708	0.40	1.22			8.5			1.2
	28	708 ~ 718	0.35	1.13			8.0			1.2
	29	718 ~ 727	0.30	1.11			8.0			1.2
	30	727 ~ 736	0.30	1.03			7.5			1.2

### Feeding management

Each treatment group was placed in a 5.0 m × 10.0 m pen (six animals per pen) and administered the assigned diets twice per day. All animals had *ad libitum* access to water. Feed intake was recorded every day, and animals were weighed every month throughout the experiment. Animals were cared and managed according to Korean traditional farm regulations.

### Meat quality measurement

At the end of the experimental period, animals were fasted for 24 h, weighed, and slaughtered at a commercial abattoir located in Ansung, Kyunggi province, Korea. Carcass measurement were obtained after chilling for 24 h at 4°C. Carcass yield and quality were graded by meat graders using the criteria provided by Livestock Quality Assessment [7].

### Evaluation of carcass chemical composition

#### a. Chemical composition

Chemical composition, including moisture, ash, crude protein, and fat contents, were analyzed according to the AOAC methodology [8]. Moisture content (%) of loin muscle samples (2 g) was measured by homogenizing and drying samples at 105°C in an oven and then measuring weight loss after drying. Total lipids were analyzed by the soxhelt extraction method. Crude protein content was measured by the Kjeldahl method. Briefly, 0.5 g of loin samples was digested at 450°C for 5 h, distilled by addition of 50% NaOH, and titrated with HCL, after which the total protein amount was calculated by multiplying% N by 6.25.

#### b. Meat color

Meat color, including Hunter L (lightness), a (redness), and b (yellowness), was determined by a Chroma Meter (CR-10, Minolta Corporation, LTD, Japan).

#### c. Melting point

Melting point was measured by the slip-point method. Briefly, lipids were extracted from meat samples by cutting them into small pieces with a Hanil Mini Cooking Cutter (Hanil electric co. HMC-150 T), homogenization with chloroform and methanol (2:1 v/v) solution, filtration, and then evaporation with nitrogen. Capillary tubes (100 mm, open) were filled to a height of 1 cm from one end and then placed in a freezer (−20°C) until lipids were firm (about 24 h). After removal from the freezer, the capillary tube was place on a warm incubator, and the temperature was increased at a rate of 1°C per min. with stirring until the lipids melted.

### Economic analysis

We analyzed the economic values of Hanwoo steers used in this experiment by calculating the average carcass prices at four different slaughter points. Profits from by-products were also considered as economic values. Feed costs for both the concentrate and roughage used in this analysis were applied as the actual purchase price of the farm where this experiment was performed. Costs for purchasing the calves, bedding, medicine, utilities (water and heating), and castration were averaged based on the number of animals used in this experiment.

### Statistics

Data was analyzed by t-test of SAS [9]. Probability values less than 0.05% were considered significant. Data of feed intake and feed conversion rate from the breeding group were excluded from the significance test.

## Results and discussion

### Performance

Changes in body weight (BW) and ADG in steers fed the experimental diets are shown in Table 6. There was no significant difference in BW or ADG between the treatments during growing (5 ~ 14 mo.) and fattening (15 ~ 22 mo.) phases. However, by finishing phase (23 ~ 30 mo.), the GC group (739.24 kg BW and 0.67 kg ADG) showed greater (P < 0.05) BW and ADG than the FC group (702.93 kg BW and 0.59 kg ADG). Consistent with this result, steers in the GC group also showed greater (P < 0.05) BW and ADG than the FC group throughout the entire experimental period (5 ~ 30 mo.). There was no significant difference in feed intake for either concentrate or roughage between the treatments during growing and fattening phases (Table 7). Feed intake for concentrate was 7.4% higher in the GC group compared to the FC group, whereas the feed conversion rate was 6.7% lower in the GC group compared to the FC group during finishing phase. The GC group showed a 3.0% greater feed intake for concentrate as well as a 3.3% lower feed conversion rate compared to the FC group for the entire experimental period. There was no significant difference in feed intake for roughage between the treatment groups. These results indicate that the physical form of concentrate have no affect on the ADG or feed intake of steers during growing and fattening phases. Zinn and Barajas [10] also reported that steers administered various densities of corn and barley for 86 d showed no difference in body weight gain or ADG. Consistent with these results, administration of various densities of sorghum flakes (412, 360, 309, and 257 g/L) did not affect the ADG of steers during growing phase [11]. However, in our study, steers from the GC group during finishing phase showed higher (P < 0.05) BW and ADG than those from the FC group. This result might be associated increased feed intake in the GC group. Furthermore, in a previous report, steers fed mashed concentrate during growing phase and then switched to flaked concentrate during finishing phase showed a greater (by 0.98 kg) ADG compared to those fed flaked concentrate for the entire period [12]. Flaking improves the feed conversion rate by inducing gelatinization of starch [13]. On the other hand, administration of flaked concentrate for the entire feeding period may decrease feed intake by reducing the rumen pH, which is associated with accelerated degradation of starch [14]. Taken together, feeding steers grounded concentrate during growing and fattening phases and then switching to flaked concentrate finishing phase effectively improved ADG and feed intake.

**Table 6:** **Body weight and daily gain of Hanwoo steers by treatment**

Items	FC^1)^	GC^2)^	T-test^3)^
No. of heads	18	24	
Body weight (kg)			
Initial (5 mo)	146.7 ± 1.35	147.4 ± 0.99	0.9253
Growing (14 mo)	370.7 ± 1.81	374.5 ± 1.22	0.6985
Fattening (22 mo)	562.2 ± 2.47	575.1 ± 1.90	0.2297
Finishing (30 mo)	702.9 ± 2.90	739.2 ± 2.67	0.0483
Average daily gain (kg)			
Growing phase	0.84 ± 0.00	0.85 ± 0.00	0.6629
Fattening phase	0.77 ± 0.01	0.80 ± 0.01	0.3220
Finishing phase	0.59 ± 0.00	0.67 ± 0.01	0.0548
Overall period	0.73 ± 0.00	0.78 ± 0.00	0.0414

^1)^Growing (flaked & pelleted diet), fattening (flaked & pelleted diet) & finishing (flaked & pelleted diet).

^2)^Growing (ground diet), fattening (ground diet) & finishing (flaked & pelleted diet).

^3)^Probability of the T test.

**Table 7:** **Feed intake and feed conversion in Hanwoo steers**

Items	FC^1)^	GC^2)^
Growing phase		
Feed intake (kg/head/day)		
Concentrate	3.52	3.53
Timothy hay	2.60	2.78
Alfalfa hay	0.68	0.70
Sub-total	3.28	3.48
Feed conversion, kg/kg	8.14	8.27
Fattening phase		
Feed intake (kg/head/day)		
Concentrate	7.91	7.89
Timothy hay	1.01	0.91
Tall fescue straw	0.80	0.99
Ryegrass straw	0.10	-
Sub-total	1.91	1.89
Feed conversion, kg/kg	12.83	12.19
Finishing phase		
Feed intake (kg/head/day)		
Concentrate	7.80	8.38
Tall fescue straw	-	1.23
Ryegrass straw	1.23	-
Sub-total	1.23	1.23
Feed conversion, kg/kg	15.39	14.38
Overall period		
Feed intake (kg/head/day)		
Concentrate	6.32	6.50
Timothy hay	1.25	1.28
Alfalfa hay	0.24	0.25
Tall fescue straw	0.26	0.72
Ryegrass straw	0.42	-
Sub-total	2.18	2.25
Feed conversion, kg/kg	11.59	11.22

^1)^Growing (flaked & pelleted diet), fattening (flaked & pelleted diet) & finishing (flaked & pelleted diet).

^2)^Growing (ground diet), fattening (ground diet) & finishing (flaked & pelleted diet).

### Carcass characteristics

Carcass weight, backfat thickness, *M. Longissimus dorsi* area, marbling score, and meat color of Hanwoo steers fed the experimental diets are shown in Table 8. Carcass weight of the GC group (4229.57 kg) was numerically, but not statistically, higher than that of the FC group (405.94 kg). There was no significant difference in backfat thickness between the treatments. *M. Longissimus dorsi* area of the GC group (91.00cm^2^) was greater (P < 0.05) than that of the FC group (83.59cm^2^). Marbling score, which is a meat quality trait, was 18.4% higher in the GC group (6.96) compared to the FC group (5.88). No difference was found in meat color, fat color, texture, or maturity between the treatments. The GC group showed a higher percentage of 1^++^ grade (43.5%) compared to the FC group (29.4%) by 48%. Percentage of quality grade over 1^+^ grade was also higher in the GC group (87%) than in the FC group (29.4%) by 50%. Brandt et al [15] also reported that supplementation of steam-flaked corn to steers increases the *M. Longissimus dorsi* area. However, carcass weight and backfat thickness are not affected by densities of flaked corn [16]. Moreover, consistent with our current results, NIAS [12] reported that administration of powdered concentrate during fattening phase followed by flaked concentrate during finishing phase to steers increased the marbling score by 13.3% compared to the flake-fed group for the entire period. In the current study, steers in the GC group showed improved *M. Longissimus dorsi* area and marbling score.

**Table 8:** **Effects of physical forms of concentrate on carcass characteristics in Hanwoo steers**

Items	FC^1)^	GC^2)^	T- test^3)^
Yield traits			
Cold carcass, kg	405.94 ± 2.08^4)^	429.57 ± 1.67	0.0542
Backfat thickness, mm	16.88 ± 0.26	17.96 ± 0.22	0.4892
Longissmus muscle area, cm^2^	83.59 ± 0.48	91.00 ± 0.44	0.0178
Yield index	62.30 ± 0.17	61.71 ± 0.14	0.5568
Yield grade,%			
A	0.0^5)^	0.0	
B	58.8	47.8	
C	41.2	52.2	
Quality traits			
Marbling score^6)^	5.88 ± 0.12	6.96 ± 0.06	0.0650
Meat color^7)^	4.88 ± 0.02	4.65 ± 0.02	0.1014
Fat color^8)^	2.94 ± 0.01	2.78 ± 0.02	0.1735
Texture^9)^	1.24 ± 0.03	1.04 ± 0.01	0.0729
Maturity^10)^	2.35 ± 0.03	2.57 ± 0.02	0.1931
Quality grade,%			
1^++^	29.4	43.5	
1^+^	29.4	43.5	
1	17.7	8.7	
2	23.5	4.3	

^1)^Growing (flaked & pelleted diet), fattening (flaked & pelleted diet) & finishing (flaked & pelleted diet).

^2)^Growing (ground diet), fattening (ground diet) & finishing (flaked & pelleted diet).

^3)^Probability of the T test. ^4)^Mean ± Standard error.

^5)^Value in parentheses represents percentage of total heads.

^6)^9 = the most abundant, 1 = devoid. ^7)^7 = dark red, 1 = bright.

^8)^7 = yellowish, 1 = white.^. 9)^3 = Coarse, 1 = fine. ^10)^9 = mature, 1 = youthful.

### Physicochemical characteristics of carcass

Effects of various physical forms of feeds on the physicochemical characteristics of Hanwoo steers are shown in Table 9. Moisture levels of the *M. Longissimus dorsi* muscle from steers in the FC and GC groups were 62.91 and 61.48%, respectively. Contents of crude protein in *M. Longissimus dorsi* muscle from steers in the FC and GC groups were 19.35 and 18.75%, respectively. Crude fat content of the GC group (18.37%) was greater than that of the FC group (15.90%) by 15.5%. The overall range of measured CIE values, including L (lightness), b (yellowness), and h (color), were greater (P < 0.05) in the GC group compared to the FC group. Physicochemical properties of meat are normally affected by moisture and crude fat content [17]. Levels of crude fat and CIE values (L) increase while moisture and crude proten content decrease with an increase in meat quality [18, 19]. Physicochemical characteristics of beef might be preferentially related to meat quality grade rather than the physical form or processing method of feed [20]. Melting point of carcass fat was highest in perirenal fat, followed by intramuscular fat and then subcutaneous fat (Table 10). Although not significant, the melting points of subcutaneous fat and intramuscular fat were lower in the GC group compared to the FC group by 4.0 and 3.0%, respectively. Carcass fat melting point was the greatest in perireral fat, followed by intramuscular fat and then subcutaneous fat. Melting point is highly correlated with fatty acid composition rather than the type or processing method of feed [21]; a higher fatty acid content is associated with a lower melting point and vice versa [22]. Taken together, the GC group with a high marbling score and meat quality grade showed higher carcass physiocochemical properties, including crude fat content and CIE.

**Table 9:** **Effects of physical forms of concentrate on physicochemical characteristics of** ***M.longissimus dorsi*** **muscle in Hanwoo steers**

Items	FC^1)^	GC^2)^	T- test^3)^
Moisture,%	62.91 ± 0.19^4)^	61.48 ± 0.17	0.2246
Crude fat,%	15.90 ± 0.28	18.37 ± 0.22	0.1240
Crude protein,%	19.35 ± 0.06	18.75 ± 0.05	0.1058
CIE value:^5)^			
L	40.43 ± 0.10	43.35 ± 0.07	0.0016
a	23.35 ± 0.06	23.81 ± 0.04	0.3895
b	11.10 ± 0.03	11.77 ± 0.02	0.0242
chroma	25.76 ± 0.06	26.60 ± 0.05	0.1604
hue	25.36 ± 0.04	26.60 ± 0.02	0.0120
Cooking loss,%	29.79 ± 0.05	28.62 ± 0.07	0.2096

^1)^Growing (flaked & pelleted diet), fattening (flaked & pelleted diet) & finishing (flaked & pelleted diet).

^2)^Growing (ground diet), fattening (ground diet) & finishing (flaked & pelleted diet).

^3)^Probability of the T test.

^4)^Means ± standard error.

^5)^L = lightness, a = redness, b = yellowness.

**Table 10:** **Effects of physical forms of concentrate on melting point of carcass fat in Hanwoo steers**

Items	FC^1)^	GC^2)^	T- test^3)^
Perirenal fat	38.82 ± 0.05^4)^	39.49 ± 0.03	0.5825
Subcutaneous fat	21.39 ± 0.03	20.54 ± 0.02	0.3142
Intramuscular fat	26.81 ± 0.05	26.02 ± 0.02	0.6595

^1)^Growing (flaked & pelleted diet), fattening (flaked & pelleted diet) & finishing (flaked & pelleted diet).

^2)^Growing (ground diet), fattening (ground diet) & finishing (flaked & pelleted diet).

^3)^Probability of the T test.

^4)^Means ± standard error.

### Economic analysis

Effects of various physical forms of concentrate on profitability are shown in Table 11. Carcass sale price for the FC and GC groups were 6,036,373 and 6,667,053 won (KRW), repectively. Carcass and by-product sale prices of the GC group were greater than those of the FC group by 10.0%. Total operating expenses, including calf purchase expenses, feed, slaughter, and other expenses, increased by 0.5% in the GC group (5,360,702 won) compared to the FC group (5,334,848 won). Gross income per head excluding operating expenses was 59.3% greater in the GC group (1,647,512 won) compared to the FC group (1,034,343 won). This increase in total revenue in the GC group can be attributed to an elevated carcass weight and percentage of meat grade above 1^+^. Total operating expenses in the GC group were 7.5% greater than those in the FC group due to increased feed intake following replacement of grounded feed by flaked concentrate during finishing phase. Operating expenses in the GC group were also higher due to elevated slaughter expenses due to increased carcass weight. In conclusion, administration of grounded concentrate during growing and fattening phases followed by flaked concentrate during finishing phase improves the profit and productivity of Hanwoo steers.

**Table 11:** **Effects of physical forms of concentrate on profits in Hanwoo steers**

Items	FC^1)^	GC^2)^
Cold carcass, kg	405.94 ± 2.08^3)^	429.57 ± 1.67
1. Gross income(A)		
Carcass sales^4)^	6,036,373.19	6,667,053.48
By-product sales^5)^	332,818	341,162
Total income	6,369,191.19	7,008,215.48
2. Operating cost(B)		
Calves	2,430,952	2,430,952
Concentrate^6)^	1,226,259.2	1,226,740.2
Roughage^7)^	576,954.3	593,850.3
Butchery expense^8)^	227,044	235,522
Self-help funds	20,000	20,000
Miscellaneous expenses^9)^	853,638	853,638
Total cost	5,334,847.5	5,360,702.5
3. Profit(A-B)	1,034,343.69	1,647,512.98

^1)^Growing (flaked & pelleted diet), fattening (flaked & pelleted diet) & finishing (flaked & pelleted diet).

^2)^Growing (ground diet), fattening (ground diet) & finishing (flaked & pelleted diet).

^3)^Means ± standard error.

^4)^Carcass price, won/kg: 1^++^B = 17,413, 1^+^B = 15,133, 1B = 14,290, 1^++^C = 16,507, 1^+^C 14,398, 1 = 13,570, 2 13,330, 2 12,370.

^5)^Includes intestines, head, legs, hide, blood, and inedible fat.

^6)^Concentrate price, won/kg : Growing(F) = 266.8, Growing(M) = 256.0, Fattening(F) = 264.0,

Fattening(M) = 253.2, Finishing(F) = 241.6

^7)^Roughage price, won/kg: Timothy hay = 407, Alfalfa hay = 360, Tall fescue straw = 240, Ryegrass straw = 240.

^8)^Butchery expense: tax, dissection operation, stamp duty, inspection & grading fee.

^9)^Miscellaneous expenses: hired labor, bedding materials, electricity, transport, water service & veterinary & medicine.

## Conclusion

This study was performed to investigate the effects of different forms of concentrate fed to Hanwoo steers on performance, carcass characteristics, and economic performance. In conclusion, it is plausible that feeding steers grounded concentrate during growing and fattening phases followed by flaked concentrate during finishing phase can improve ADG and feed intake. This feeding strategy increases the profit and productivity Hanwoo steers.

## References

[1] Jeong J, Jin GL, Shinekhuu J, Ji BJ, Song MK (2009). Effect of method on nutrient availability in ruminants. Bull Biotechnol.

[2] Hale WH, Cuitun L, Saba WJ, Taylor B, Theurer B (1966). Effect of steam processing and flaking milo and barley on performance and digestion by steers. J Anim Sci.

[3] Han YK, Maeng WJ, Park GK, Paik IK, Ohh SJ, Choi YJ (1993). Feed Processings.

[4] Theurer CB, Lozano O, Alio A, Delgado-Elorduy A, Sadik M, Huber JT, Zinn RA (1999). Steam-processed corn and sorghum grain flaked at different densities alter ruminal, small intestinal, and total tract digestibility of starch by steers. J Anim Sci.

[5] Neock JE, Tamminga S (1991). Site of digestion of starch in the gastrointestinal tract of dairy cows and its effect on milk yield and composition. J Dairy Sci.

[6] Owens FN, Secrist DS, Hill EJ, Gill DR (1997). The effect of grain source and grain processing on performance of feedlot cattle: a review. J Anim Sci.

[7] Livestock Quality Assessment (2007). Grading of Carcasses of Methods, Standards and Enforcement.

[8] AOAC (1990). Official Methods of Analysis.

[9] SAS (2002). SAS User's Guide:Statistics.

[10] Zinn RA, Barajas R (1997). Influence of flake density on the comparative feeding value of a barley-corn blend for feedlot cattle. J Anim Sci.

[11] Swingle RS, Eck TP, Theurer CB, De la Llata N, Poore MG, Moore JA (1999). Flake density of steam-processed sorghum grain alters performance and sites of digestibility by growing-finishing steers. J Anim Sci.

[12] National Institue of Aniamal Science (1998). Development of technologies for production of high-quality beef from Hanwoo steers. Effects of Physical Form of Concentrate on Productivity of Hawoo by Feeding Period.

[13] Theurer CB (1986). Grain processing effect on starch utilization by ruminants. J Anim Sci.

[14] Ministry of Ariculture, Food and Rural Affairs (2005). Development of technologies to improve competitiveness of Hanwoo. Development of feeding program for production of high-quality beef from Hanwoo steers.

[15] Brandt RT, Kuhl GL, Campbell RE, Kastner CL, Stroda SL (1992). Effects of steam-flaked sorghum grain or corn and supplemental fat on feedlot performance, carcass traits, longissimus composition, and sensory properties of steers. J Anim Sci.

[16] Hales KE, McMeniman JP, Leibovich J, Vasconcelos JT, Quinn MJ, May ML, DiLorenzo N, Smith DR, Galyean ML (2010). Effects of varying bulk densities of steam-flaked corn and dietary roughage concentration on in vitro fermentation, performance, carcass quality, and acid–base balance measurements in finishing steers. J Anim Sci.

[17] Kelly RF, Fontenot JP, Graham PP, Wilkinson WS, Kineaid CM (1968). Estimates of carcass composition of beef cattle fed at different plane of nutrition. J Anim Sci.

[18] Savell JW, Cross HR, Smith GC (1986). Percentage ether extractable fat and moisture content of beef longissimus muscle as related to USDA marbling score. J Food Sci.

[19] Nelson JL, Dolezal HG, Ray FK, Morgan JB (2004). Characterization of certified angus beef steaks from the round, loin, and chuck. J Anim Sci.

[20] Cameron PJ, Zembayashi M, Lunt DK, Mitsuhashi T, Mitsumoto M, Dzawa S, Smith SB (1994). Relationship between Japanese Beef Marbling Standard and intramuscular lipid in the m. longissimus thoracis of Japanese Black and American Wagyu cattle. Meat Sci.

[21] Wood JD, Wiseman J (1984). Fat deposition and the quality of fat tissue in meat animals. Fats in Animal Nutrition.

[22] Enser M, Wood JD (1993). Proceeding of the 39th lnternational congress of meat Science and Technology. Effect of Time of Year on Fatty Acid Composition and Melting Point of UK Lamb.

